# The London Hospital

**Published:** 1903-01-17

**Authors:** 


### THE LONDON HOSPITAL.

### QUINQUENNIAL APPEAL FOR HALF A MILLION.

The Hon. Sydney Holland, Chairman of the London Hospital, has issued an urgent appeal in favour of this great institution. He points out that this hospital only begs widely every five years, and that this year is their quinquennial, which means that it is necessary to get enough now to carry on the work for the next five years, and on this occasion to pay also for the improvements they have been compelled to make.

After eloquently describing the work and the wants of the hospital he continues :—

I do not want to weary those who are good enough to read this letter with many figures, but I should like to say that the hospital is not at present in debt. We have had to sell some investments, and, unless very largely helped, shall have to sell over £100,000 more in the course of the next two years.

We have about £257,000 invested, and our income from this, from trust funds, and from our estate is only £22,000 a year. It takes £85,000 a year to keep up the hospital. Of course, this sum seems enormous and impossible to the man who is feeling in his pocket to see if he has a threepenny bit instead of a sixpence, while singing lustily—

> "Were the whole realm of nature mine,
> This were an offering far too small."

But the public have been in the past very generous to the London Hospital; and I am loth to believe that so long as we do the work honestly, wholeheartedly, and economically we shall ever be compelled to cripple the work for want of money, though I confess that the outlook is very black just now, when the hospital is poorer than it has been in its 150 years of existence.

Here is no question of a hospital situated where it is not wanted. The most ardent hospital reformer has never ventured to suggest that the London Hospital could be moved one foot away either north, east, south, or west from its present site. It is in the midst of the poorest people on God's earth, and is the hospital for the Port of London and for most of the riverside workers.

Here is no question of a hospital with large invested funds or endowments. I suppose for its size the "London" has less money invested than any hospital in England, and even of this we have had to sell some portion.

Despite all its disadvantages, the hospital has made great strides since I last appealed to the public five years ago. An isolation block has been built (there was none before) at a cost of £32,000, the gift of an anonymous donor. Another anonymous donor has given us five of the finest operating theatres in the world at a cost of £13,000. We have built and opened a large pathological department at a cost of £20,000, which Mr. C. Morrison has endowed for three years. We have built a new out-patient department costing £70,000, to which an anonymous donor has given £25,000, where at last we shall be able to treat these poor people properly. We have had a convalescent home built for us by the executors of the late Baroness de Stern, and Mr. Hora has endowed a women's ward to be built.

This year their Majesties the King and Queen have graciously intimated their intention of coming down to open the new out-patient block, and we are very anxious to show them that their great kindness in doing this will be of material assistance to the hospital.

That we are now under the necessity of again appealing to the public will, I trust, have been made abundantly clear by the figures which I have set out in this letter. I feel confident that the present appeal for help will meet with the same generous recognition and response as have our previous quinquennial appeals, and that we shall thereby be able to continue and maintain with the same efficiency the work of this great hospital.

